# Waveform Optimization for Target Estimation by Cognitive Radar with Multiple Antennas

**DOI:** 10.3390/s18061743

**Published:** 2018-05-29

**Authors:** Yu Yao, Junhui Zhao, Lenan Wu

**Affiliations:** 1 School of Information Engineering, East China Jiaotong University, Nanchang 330031, China; junhuizhao@bjtu.edu.cn; 2 School of Information Science and Engineering, Southeast University, Nanjing 210096, China; wuln@seu.edu.cn

**Keywords:** cognitive radar system, Kalman filtering, temporal correlated target, multiple antennas, waveform optimization

## Abstract

A new scheme based on Kalman filtering to optimize the waveforms of an adaptive multi-antenna radar system for target impulse response (TIR) estimation is presented. This work aims to improve the performance of TIR estimation by making use of the temporal correlation between successive received signals, and minimize the mean square error (MSE) of TIR estimation. The waveform design approach is based upon constant learning from the target feature at the receiver. Under the multiple antennas scenario, a dynamic feedback loop control system is established to real-time monitor the change in the target features extracted form received signals. The transmitter adapts its transmitted waveform to suit the time-invariant environment. Finally, the simulation results show that, as compared with the waveform design method based on the MAP criterion, the proposed waveform design algorithm is able to improve the performance of TIR estimation for extended targets with multiple iterations, and has a relatively lower level of complexity.

## 1. Introduction

Cognitive radars have received a lot of attention in recent years. Similar to brain-empowered system architectures, cognitive radar employs adaptive feedback principle to facilitate adaptive detection of the time-invariant target scene. Subsequently, the target feature information in the backscatter signal is exploited to allocate the power or spectrum of the probing signal at the transmitter [1,2]. Cognitive radar usually forms a closed feedback loop from the receiver to the transmitter. It is able to adaptively adjust probing signals or the receiver to suit the time variant target scene. The feedback loop has great potentials in improving the performance of target recognition and detection, as shown in [3,4].

The transmitted waveforms of the cognitive radar are constantly adjusted in order to extract target feature information in a time variant environment. Many methods to design the cognitive waveform have been proposed, in several recent works. The literature [5] developed waveform design methods that provide good performance for target resolution. Bell [6] discussed the waveform design problem for extended target detection and parameter estimation. Since the received signals feature clutter and noise interference, several waveform design algorithms are proposed which maximize signal to noise ratio (SNR) and signal to interference plus noise ratio (SINR) of the output signal [7,8,9,10]. The cognitive radar waveform is optimized by maximizing the probability of target detection, instead of the SINR of the output signal [11,12]. The corresponding algorithm is also discussed in the literature [13]. Under the condition of given transmitted power constraint, the water-filling method is presented to allocate the transmitted power, and maximize the mutual information [14,15,16,17,18]. Cognitive waveform design has been studied as a means of improving the performance of target estimation and identification [19,20,21,22,23]. For instance, the literature [24] proposes that the mutual information (MI) between the echo waveform and the estimate of TIR be minimized. The literature [25] proposes minimizing the mean square error (MSE) of the estimate of TIR. Kalman filtering-based method is proposed by F. Z. Dai [26] to exploit target temporal correlation. Compared with the algorithms in the literature, the proposed method has good performance of target parameter estimation. However, the simulation results show that the algorithm has low reliability and high complexity, due to the convolution operation in the time domain. The Fourier transform of TIR is denoted by target scattering coefficients (TSC). The TSC estimation was applied for the adaptive waveform optimization design, with the purpose of enhancing the target detection performance [27,28,29,30]. However, TSC varies continuously with the relative movement of the target scene [31]. From the perspective of cognitive radar, the TSC estimation should be updated at the receiver, and sent to the transmitter through a feedback loop.

Recent researches have also indicated that multiple antennas radar can take full advantage of spatial diversity gains by detecting the extended target in different directions [32,33,34,35]. The multiple antennas radars are able to excite many separate scattering centers by emitting probing signals and enriching the target feature information in the backscatter signal. Since radar echoes are strongly correlated to target scattering in the line-of-sight (LOS) directions, the spatial diversity offered by multiple antennas radar system improves the capacity of target feature extraction, as presented in literature [36]. From the literature [37], one pulse optimization design scheme involves applying the principle of information theory to radar signal processing. Following the pioneering work of Bell [6], information-theoretic measures was applied for the adaptive waveforms design with the purpose of enhancing the radar detection and classification performance. The spatial diversity, which is provided by different paths and angles of echo waveforms, is utilized to improve radar efficiency [38,39]. Yang and Blum [40,41] extended the Bell’s work by using mutual information (MI) as the waveform optimization criterion, subject to limited transmission power in the multiple antennas radar configuration.

In this paper, we analyze the performance of an adaptive multi-antenna radar system that contains the idea of “Kalman Filtering” and “TIR estimation”. A novel method based on Kalman filtering optimizing the waveforms of an adaptive multi-antenna radar system for TIR estimation is presented. The receiver adopts the Kalman Filtering approach by updating the target parameters. The radar system updates the TIR estimation and utilizes this information to choose the optimal waveform for transmission. An adaptive feedback loop enables the delivery of the estimated value of TIR to the transmitter. The transmitter adapts its transmitted waveform to suit the time-invariant environment. The proposed waveform design algorithm is studied in order to improve the performance of TIR estimation, which can be summarized as follows:

(1) We extract the target feature information derived from successive received signals at the receiver. The TIR can be considered as a temporally-correlated function during pulse repetition interval (PRI).
(2) By utilizing this temporal correlation of the TIR changes, the waveform design problem is modeled by minimizing the MSE of TIR estimation. The MSE of TIR estimation can be obtained during the Kalman Filtering-based iteration process.

The main contributions of this paper are summarized as follows:

(1) We present an adaptive multi-antenna radar system model, based on the idea of the MSE of TIR minimization.
(2) We present a Kalman filtering-based waveform design approach by making use of temporal correlation of the TIR derived from successive received signals.
(3) We provide performance analysis of the adaptive multi-antenna system in terms of the TIR estimation by the proposed iteration steps. The proposed algorithm has relatively lower complexity, due to the product operation in the time domain.

The structure of this paper is as follows. In Section 2, an adaptive multiple antennas radar system model for extended target is formulated. In Section 3, TIR estimation based on maximum a posteriori (MAP) criterion is discussed. The Kalman Filtering-based approach is developed to make use of the temporal correlation of the TIR. In Section 4, by utilizing the temporal correlation of the TIR changes, a waveform design algorithm based on Kalman filtering-based approach is developed by minimizing the MSE of TIR estimation. A method is proposed to find the optimal solutions for the optimization problem. The simulation results illustrating the proposed methods are provided in Section 5. The conclusions are given in Section 6.

Throughout this paper, the following notations will be used. Vectors are denoted by boldface lowercase letters and matrices by boldface uppercase letters. ${\left(\cdot \right)}^{H}$ and ${\left(\cdot \right)}^{T}$ denote transpose conjugate operation and the transpose, respectively. diag{.} denotes the diagonal matrix. Γ is the Fourier transform. Tr{.} is the trace of a matrix. ∗ is linear convolution operator, E{ } is expectation operator and Var{·} is variance operator. The symbol “∘” describes the Hadamard product.

## 2. The Model of the Multiple Antennas Radar System

We consider that an adaptive multi-antenna radar system is equipped with a transmit array and a receive array comprising N antennas and Q antennas, respectively. To simplify our modeling, we assume that N=Q. In this work, we assume that two different antennas in the multi-antenna radar system are independent. The distance between the two antennas are sufficient and known. The transmit array antennas send probing signals to the target environment, and the backscattered signals are received at the receive array antennas. The characteristics of the scattering field are examined for target information extraction and detection.

**Figure 1:**
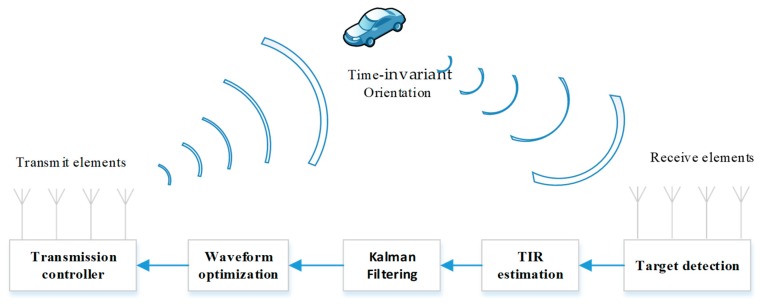
Adaptive multi-antenna radar architecture.

In the intelligent transportation scenario, we analyze the performance of an adaptive multi-antenna radar system that contains the idea of “Kalman filtering” and “TIR estimation”. The system architecture of the adaptive multi-antenna radar is presented in Figure 1. The system consists of four modules: transceiver device; TIR estimation module; Kalman filtering module; waveform optimization module. The Kalman filtering module is a novel scheme that distinguishes the proposed adaptive system from a traditional feedback system.

In this work, we assume that the target was contained in a single range cell, which consists of M scattering centers located at $\theta_{m}=\left\{x_{m},y_{m}\right\},\left(m=1,2,\ldots ,M\right)$ with reflectivity $a_{m}^{2}$. The target is illuminated by N transmitters located at $\theta_{T,n}=\left\{x_{n},y_{n}\right\},\left(n=1,2,\ldots ,N\right)$. The backscattering signals are collected by Q receivers located at $\theta_{R,q}=\left\{x_{q},y_{q}\right\},\left(q=1,2,\ldots ,Q\right)$. Next, we utilize a set of transmitted waveforms $f_{n},n=1,\ldots ,N$ for illumination, where $\int_{T_{p}}{\left|f_{n}\right|}^{2}=1$. $T_{p}$ denotes the waveform duration. The backscattering signal observed at the *q*-th receiver, due to the probing signal sent from the *n*-th transmitter and reflected from the *m*-th scatterer (excluding noise), is denoted as:

(1) $$r_{n,q}^{m}=h_{n,q}^{m}f_{n}(t-\tau_{T,n}\left(\theta_{m}\right)-\tau_{R,q}\left(\theta_{m}\right))$$

where $h_{n,q}^{m}=a_{m}^{2}\exp\left\{-j2\pi f_{c}\left[\tau_{T,n}\left(\theta_{m}\right)+\tau_{R,q}\left(\theta_{m}\right)\right]\right\}$, $h_{n,q}^{m}$ is the TIR from the *n*-th transmitter to the *q*-th receiver via the *m*-th scatterer. $\tau_{T,n}\left(\theta_{m}\right)$ and $\tau_{R,q}\left(\theta_{m}\right)$ are the path delays from the *n*-th transmitter to the *m*-th scatterer, and from the *m*-th scatterer to the *q*-th receiver, respectively. It is worth noting that the target moves with a constant velocity. As the radar carrier, Doppler is not useful for waveform optimization design; this is ignored in this paper.

In order to simplify the discussion, we assume that the target has a center of gravity at $\theta_{0}=\left\{x_{0},y_{0}\right\}$, that is, $f_{n}(t-\tau_{T,n}\left(\theta_{m}\right)-\tau_{R,q}\left(\theta_{m}\right)=f_{n}(t-\tau_{T,n}\left(\theta_{0}\right)-\tau_{R,q}\left(\theta_{0}\right)$ for all m=1,…,M. Therefore, the above Equation (1) can be rewritten as:

(2) $$r_{n,q}=h_{n,q}f_{n}(t-\tau_{T,n}\left(\theta_{0}\right)-\tau_{R,q}\left(\theta_{0}\right))$$

where $h_{n,q}=\sum_{m=1}^{M}h_{n,q}^{m}$. $h_{n,q}$ is the TIR from *n*-th transmitter to the *q*-th receiver via the target. The vector version of the backscattering signal at *q*-th receiver can be expressed as:

(3) $$r_{q}=\sum_{n=1}^{N}h_{n,q}{\hat{f}}_{n,q}+n_{q}$$

The term ${\hat{f}}_{n,q}\in {\mathbb{R}}^{K\times 1}$, where $K=K_{n}+K_{d}$. $K_{n}$ is the length of the transmitted signal and $K_{d}$ is the maximum delay with respect to the first arrival among all the links. ${\hat{f}}_{n,q}={\left[\begin{array}{ccc} 0_{1\times L_{n,q}} & f_{n}^{T} & 0_{1\times (K_{d}-L_{n,q})} \end{array}\right]}^{T}$, where $f_{n}\in {\mathbb{R}}^{K_{n}\times 1}$, 0 is a null vector, $L_{n,q}$ is the delay from the *n*-th transmitter to the *q*-th receiver via the target. $n_{q}$ denotes additive white Gaussian noise (AWGN) at the *q*-th receiver. We further assume the scenario that $K_{n}$ is much larger than $K_{d}$. As a result, $f_{n}\approx {\hat{f}}_{n,q}$.

At time k, we consider $R_{k}=\left[\begin{array}{cccc} r_{k,1} & r_{k,2} & \ldots & r_{k,Q} \end{array}\right]$ and $F_{k}=\left[\begin{array}{cccc} f_{k,1} & f_{k,2} & \ldots & f_{k,N} \end{array}\right]$ are the matrices of received signals and transmitted signals, respectively; $H_{k}={\left[h_{n,q}^{\left(k\right)}\right]}_{N\times Q}$ and $N=\left[\begin{array}{cccc} n_{1} & n_{2} & \ldots & n_{N} \end{array}\right]$ are the TIR matrix and AWGN matrix, respectively. At the result, if the target is present, the matrices version of the backscattering signals at time k disturbed by the AWGN can be expressed as follows:

(4) $$R_{k}=F_{k}H_{k}+N$$

where k is the index of radar pulse sample. $H∼\mathbb{C}N\left(0,C_{T}\right)$ and $N∼\mathbb{C}N\left(0,C_{N}\right)$. $C_{T}$ is the covariance matrix of TIR and $C_{N}$ is the covariance matrix of AWGN. We consider that H and N are independent of each other. The total transmitted energy is denoted by $\frac{1}{N}\sum_{n=1}^{N}{\left|f_{n}\right|}^{2}=E_{f}$. The multiplication operation in physical space for waveform design can be obtained, which facilitates the application of many mathematical natures and principles. The complexity of the waveform design is decreased, and the waveform optimization problem can be solved efficiently.

The relative angle between the multi-antenna radar and the target is time-invariant during the PRI. Since we will consider the waveform design problem for target parameter estimation in the time variant radar scene, the dynamic model of the TIR should be established. From the literature [8], the TIR of different time in a short interval are correlated, and the correlation coefficient decreases with increasing time interval. During the *k*-th pulse, the time dynamic characteristic of the TIR can be described as:

(5) $$H_{k}=e^{-T/\lambda }H_{k-1}+U$$

where T denotes the radar pulses interval, and λ denotes the temporal correlation of target impulse during PRI, which is determined by the change rate of the target angle. We consider that TIR is second-order stationary process. U is the noise matrix, which is complex Gaussian noise with zero-mean and covariance matrix $e^{-2T/\lambda }C_{T}$.

We aim to explore the waveform optimization for an adaptive multi-antenna radar system. By employing the spatial diversity technology, the multi-antenna systems can improve the parameter estimation capability and target detection capability. From the literature [25], the TIR estimation is the precondition for the target detection and target classification. In this work, a novel waveform design scheme is studied, in order to improve the performance of TIR estimation, which can be summarized as follows.

In the first step, the received signals are used to extract the TIR via the Kalman filtering-based approach. The MSE matrix of TIR estimation can be obtained during the Kalman filtering-based iteration process.

In the second step, TIR is considered as a temporally-correlated function during pulse repetition interval (PRI). By utilizing this temporal correlation of the TIR changes, the waveform design problem is modeled by minimizing the MSE of TIR estimation.

**Figure 2:**
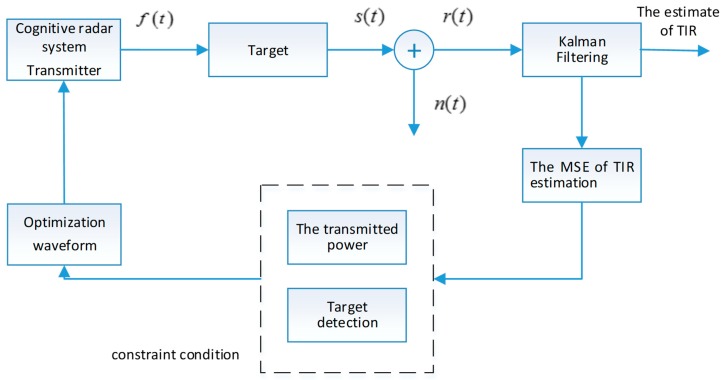
The process of waveform optimization for TIR estimation.

The cognitive processing is summarized as follows: The receiver employs the Kalman filtering approach update the target parameters. The radar system updates TIR estimation and utilizes this information to choose the optimal waveform for transmission. An adaptive feedback loop enables the delivery of estimated value of TIR to the transmitter. The transmitter adapts its probing signals to suit the time-invariant environment. The process of waveform optimization for the target parameter estimation is depicted in Figure 2.

## 3. TIR Estimation Based on MAP Criterion

In this section, we intend to estimate TIR in order to improve the performance of target detection in the adaptive multi-antenna radar system. During the *k*-th pulse sample, TIR estimation based on maximum a posteriori (MAP) criterion can be written as:

(6) $$\begin{array}{cc} {\hat{H}}_{k} & =\arg\underset{H_{k}}{\max}\;p\left(H_{k}|R_{k}\right)\\ & =\arg\underset{H_{k}}{\max}\;\frac{p\left(R_{k}|H_{k}\right)p\left(H_{k}\right)}{p\left(R_{k}\right)}\\ & =\arg\underset{H_{k}}{\max}\;{\left(R_{k}-F_{k}H_{k}\right)}^{H}C_{N}^{-1}\left(R_{k}-F_{k}H_{k}\right)-H_{k}C_{T}^{-1}H_{k}\\ & =\arg\underset{H_{k}}{\max}\;f\left(H_{k}\right) \end{array}$$

where

(7) $$\begin{array}{l} p\left(R_{k}|H_{k}\right)=\frac{1}{{\left(2\pi \right)}^{M/2}{\left|C_{N}\right|}^{1/2}}\exp\left(-\frac{1}{2}{\left(R_{k}-F_{k}H_{k}\right)}^{H}C_{N}^{-1}\left(R_{k}-F_{k}H_{k}\right)\right)\\ p\left(H_{k}\right)=\frac{1}{{\left(2\pi \right)}^{M/2}{\left|C_{T}\right|}^{1/2}}\exp\left(-\frac{1}{2}{\left(H_{k}\right)}^{H}C_{T}^{-1}H_{k}\right)\\ f\left(H_{k}\right)={\left(R_{k}\right)}^{H}C_{N}^{-1}F_{k}H_{k}+{\left(F_{k}H_{k}\right)}^{H}C_{N}^{-1}R_{k}-{\left(F_{k}H_{k}\right)}^{H}C_{N}^{-1}F_{k}H_{k}-{\left(H_{k}\right)}^{H}C_{T}^{-1}H_{k} \end{array}$$

The received waveform $R_{k}$ follows complex Gaussian distribution and $p\left(H_{k}|R_{k}\right)$ is the probability distribution of TIR. Then, the estimate of TIR can be obtained as follows:

(8) $$\frac{\partial f\left(H_{k}\right)}{\partial H_{k}}=0$$

We have

(9) $$\left[{\left(F_{k}\right)}^{H}C_{N}^{-1}F_{k}+C_{T}^{-1}\right]H_{k}={\left(F_{k}\right)}^{H}C_{N}^{-1}R_{k}$$

After the simplification, TIR estimation based on MAP criterion in AWGN channel can be written as:

(10) $${\hat{H}}_{k}=Q_{k}R_{k}$$

We have

(11) $$Q_{k}={\left[{\left(F_{k}\right)}^{H}C_{N}^{-1}F_{k}+C_{T}^{-1}\right]}^{-1}{\left(F_{k}\right)}^{H}C_{N}^{-1}$$

The MSE matrix of TIR estimation based on MAP criterion can be expressed as:

(12) $$\begin{array}{cc} e_{k} & =E\left\{{\left\|{\hat{H}}_{k}-H_{k}\right\|}_{2}^{2}\right\}\\ & =Q_{k}\left(F_{k}C_{T}{\left(F_{k}\right)}^{H}+C_{T}\right){\left(Q_{k}\right)}^{H}-Q_{k}F_{k}C_{T}-C_{T}{\left(F_{k}\right)}^{H}{\left(Q_{k}\right)}^{H}+C_{T} \end{array}$$

The temporal correlation characteristic of TIR cannot be utilized by using MAP criterion in the process of calculating estimation of parameters. In this work, we present a Kalman filtering-based method for TIR estimation under multiple antennas scenario. This method has two steps: firstly, we utilize the MAP criterion to initialize the estimated parameters, i.e., the observed state of TIR ${\hat{H}}_{1}=Q_{1}R_{1}$ and the MSE matrix of TIR estimation $P_{1/1}=e_{1}=0$. The TIR state transition and the observation processes are denoted by the above Equations (4) and (5), respectively. The MAP criterion is utilized for mapping the received signals into the observed state of TIR. The observation model is defined by $Q_{k}R_{k}$. The Kalman filtering-based iteration process can be described in Algorithm 1.
**Algorithm 1.** Kalman filtering for TIR estimation in cognitive radar system**Step 1:** Initializing iteration index k=1 and the MSE matrix of TIR estimation $P_{1/1}=0$ **Step 2:** The predicted matrix of TIR can be expressed as $${\hat{H}}_{k|k-1}=e^{-T/\lambda }{\hat{H}}_{k-1|k-1}$$
**Step 3:** The predicted MSE matrix of TIR can be obtained as $P_{k|k-1}=e^{-2T/\lambda }P_{k-1|k-1}+\left(1-e^{-2T/\lambda }\right)C_{T}$
**Step 4:** The Kalman gain matrix can be expressed as $\Phi_{k}=P_{k|k-1}{\left(Q_{k}F_{k}\right)}^{H}\left[\left(Q_{k}F_{k}\right)P_{k|k-1}{\left(Q_{k}F_{k}\right)}^{H}+Q_{k}C_{N}{\left(Q_{k}\right)}^{H}\right]$
**Step 5:** The estimated matrix of TIR can be expressed as ${\hat{H}}_{k|k}={\hat{H}}_{k|k-1}+\Phi_{k}{\hat{H}}_{k}$
**Step 6:** The MSE matrix of TIR is updated as $P_{k|k}=P_{k|k-1}-\Phi_{k}Q_{k}F_{k}P_{k|k-1}$
If $k=K_{\max}$, the process ends; otherwise, we need to go back to Step 2 and repeat.

## 4. Waveform Optimization for TIR Estimation

As extensively discussed in the existing literature [42,43], the adaptive multi-antenna radar makes use of target information from the radar echoes to improve the performance of TIR estimation. In this section, we intend to further improve the performance of TIR estimation for target detection. We develop a waveform optimization algorithm by minimizing the MSE of TIR estimation under multiple antennas scenario. During the *k*-th pulse, the MSE matrix of TIR estimation via Kalman filtering-based approach is preliminary described as follows:

(13) $$f\left(F_{k}\right)≜Tr\left\{P_{k|k}\right\}$$

Under transmitted power and detection probability constraints, the above waveform design problem (13) can be described as:

(14) $$\begin{array}{c} \underset{F_{k}}{\min}f\left(F_{k}\right)\\ \;s.t.\;{\left\|F_{k}\right\|}_{2}^{2}\leq E_{f}\\ \;P_{d}\geq \varepsilon \end{array}$$

where $E_{f}$ describes the power of transmitted waveform, $P_{d}$ represents the probability of detection. From the literature [44], we can utilize the Woodbury identity to simplify the objective function. The objective function in (14) can be expressed by the trace of the MSE matrix of TIR estimation. Hence, the above waveform design problem (14) is rewritten as the following expression:

(15) $$\begin{array}{l} F_{k}=\underset{F_{k}}{\min}Tr\left\{{\left[{\left(P_{k|k-1}\right)}^{-1}+C_{T}^{-1}-{\left(C_{T}+C_{T}{\left(F_{k}\right)}^{H}C_{N}^{-1}F_{k}C_{T}\right)}^{-1}\right]}^{-1}\right\}\\ \;s.t.\;{\left\|F_{k}\right\|}_{2}^{2}\leq E_{f}\\ \;P_{d}\geq \varepsilon \end{array}$$

As we can see from the above waveform design problem (15), the above waveform optimization problem is a convex one under AWGN channel. However, the proposed scheme is a non-convex and non-linear optimization problem, considering the clutter and jamming. It is impossible to find the theoretical solution by using an algorithm with polynomial complexity. The goal of this paper is to obtain the optimal (or sub-optimal) solutions of the above waveform design problem (15).

We propose a two-step approach to solve the above problem (15), which is based on the methods of semi-definite programming. Firstly, according to the literature [36], the above waveform design problem (15) can be considered as a convex under AWGN channel. We can obtain the optimal solution (optimization waveform) directly by using the MATLAB optimization toolbox, such as CVX [45]. Secondly, we denote the rank of the matrix $F_{k}$ as $rank\left(F_{k}\right)$. We can obtain the sub-optimal solution by solving the rank of the matrix $F_{k}$. If $rank\left(F_{k}\right)=1$, the initial waveform $f_{k}$ is the optimal solution. If $rank\left(F_{k}\right)>1$, according to the literature [32], the radar system employs the eigenvector $w_{\max}$ of the matrix $F_{k}$, corresponding to its largest eigenvalue as the reference vector. Based on the gradient method mixed by steepest decent method and Newton method, a local optimal solution (also called sub-optimal solution) can be obtained. Hence, the objective function in above Equation (15) can be rewritten as:

(16) $$\begin{array}{ll} f\left(F_{k}\right) & =Tr\left(P_{k|k}\right)\\ & =Tr\left\{P_{k|k-1}-P_{k|k-1}{\left(F_{k}\right)}^{H}{\left[C_{N}+F_{k}P_{k|k-1}{\left(F_{k}\right)}^{H}\right]}^{-1}F_{k}P_{k|k-1}\right\}\\ & =Tr{\left[{\left(P_{k|k-1}\right)}^{-1}+{\left(F_{k}\right)}^{H}C_{N}^{-1}F_{k}\right]}^{-1} \end{array}$$

where ${\left(F_{k}\right)}^{H}C_{N}^{-1}F_{k}$ can be denoted by ${\left(F_{k}F_{k}^{H}\right)}^{H}\circ N$. We have:

(17) $$f\left(F_{k}\right)=Tr{\left[{\left(P_{k|k-1}\right)}^{-1}+{\left(F_{k}F_{k}^{H}\right)}^{H}\circ N\right]}^{-1}$$

The symbols “∘” describes the Hadamard product and $N≜\left(\begin{array}{ccc} C_{N,1,1}^{-1} & \ldots & C_{N,1,Q}^{-1}\\ \ldots & \ldots & \ldots \\ C_{N,N,1}^{-1} & \ldots & C_{N,N,Q}^{-1} \end{array}\right)$. The problem of waveform design in this scheme is a non-convex non-linear optimization problem. We can convert the above optimization problem into a convex quadratic programming problem by using the method of SDP. We have:

(18) $$\begin{array}{l} T_{k}=\underset{T_{k}}{\arg\min}\left\{Tr{\left[{\left(P_{k|k-1}\right)}^{-1}+T_{k}\circ N\right]}^{-1}\right\}\\ \;s.t.\;\mathrm{Tr}\left\{T_{k}\right\}\leq E_{f}\\ \;P_{d}\geq \varepsilon \end{array}$$

where $T_{k}≜{\left(F_{k}F_{k}^{H}\right)}^{H}$. The above program (18) can be expressed as SDPs, and via hierarchies of SDPs the solutions of polynomial optimization problems can be approximated. Then, we solve a convex quadratic programming problem by the optimization theory and method constructing algorithms. The likelihood estimation can be equivalently transformed into:

(19) $$l\left(R_{k}\right)=R_{k}^{H}C_{N}^{-1}F_{k}{\hat{H}}_{k}\overset{H_{1}}{\underset{H_{2}}{≶}}T$$

where T describes the detection threshold. The probability of target detection can be rewritten as:

(20) $$P_{d}=Q\left(Q^{-1}\left(P_{fa}\right)-{\left(F_{k}{\hat{H}}_{k}\right)}^{H}C_{N}^{-1}F_{k}{\hat{H}}_{k}\right)$$

Q(.) represents the *Q*-function and $P_{fa}$ describes the probability of false alarm, which can be expressed as:

(21) $$P_{fa}=P\left(R_{k}^{H}C_{N}^{-1}F_{k}{\hat{H}}_{k}\geq T\right)$$

As the number of Kalman filtering iterations increases, the estimated value of TIR is close to the real measured value. Since *Q*-function describes a monotonic decreasing and continuous function, the expression $P_{d}\geq \varepsilon$ can be rewritten as:

(22) $$F_{k}^{H}{\hat{H}}_{k}^{H}C_{N}^{-1}{\hat{H}}_{k}F_{k}\geq \varepsilon^{\prime }$$

From the literature [36], an algorithm with polynomial complexity was presented to solve the convex quadratic programming problem. The eigenvector of the matrix $T_{k}^{\prime }$, corresponding to its largest eigenvalue, is expressed by $v_{\max}$. Then, the optimal solution $T_{k}^{\prime }$ of the above convex quadratic programming problem (18) can be obtained by the optimization toolbox. Therefore, the eigen-decomposition of the matrix $T_{k}^{\prime }$ can be utilized to obtain the optimization waveform, which can be written as:

(23) $${\hat{f}}_{n}=\frac{\sqrt{E_{f}}v_{\max}}{{\left\|v_{\max}\right\|}_{2}},n=1,\ldots ,N$$

## 5. Simulation Results

We assume that the transmitted powers of all of N transmit antennas are the same for the initial state. The process of waveform optimization for TIR estimation is depicted in Figure 2. In this section, we analyze the performance gain of TIR estimation for optimization waveforms designed by the proposed scheme. The simulation parameters are reported in Table 1.

We use the normalized MSE to define the estimation performance:

(24) $$MSE_{n}=\frac{{\left\|\hat{H}-H\right\|}_{2}^{2}}{{\left\|H\right\|}_{2}^{2}}$$

where $\hat{H}$ and H denote estimated value and the true value of TIR, respectively. During the *k*-th pulse sample, we assume that $C={\left[c_{n,q}\right]}_{N\times Q}$ is clutter impulse response (CIR) matrix, $c_{n,q}$ represent the CIR between *n*-th transmit antenna and the *q*-th receive antenna. $C∼\mathbb{C}N\left(0,C_{c}\right)$, $C_{c}$ is the covariance matrix of CIR.

**Figure 3:**
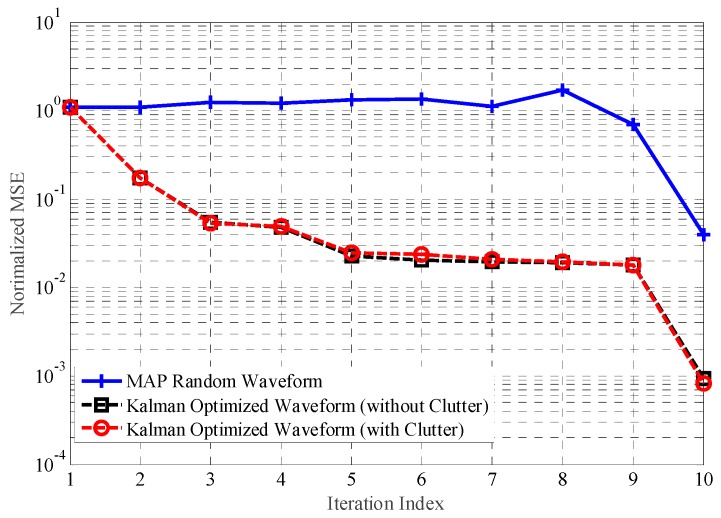
The estimation performance without the interference.

We compare the performance gain of TIR estimation for optimization waveforms designed by the proposed scheme to the gain for random waveform, and compare this result with the optimized waveform based on MAP criterion. Firstly, we consider that the backscatter signals are subject to interference from white Gaussian noise. Figure 3 presents the normalized MSE with regard to TIR estimation under the transmitted power and detection probability constraints. Eight hundred simulations have been run for each, at a particular value of the received SNR.

As the Kalman filtering approach utilizes the temporal correlation of the TIR during the pulse interval, the proposed radar system adapts its probing signal better than waveforms based on MAP criterion to the fluctuating target radar cross section (RCS). On the other hand, optimized waveforms based on MAP criterion are unable to match the time-varying TIR after multiple iterations. Hence, the TIR estimation performance is suboptimal in this case.

**Figure 4:**
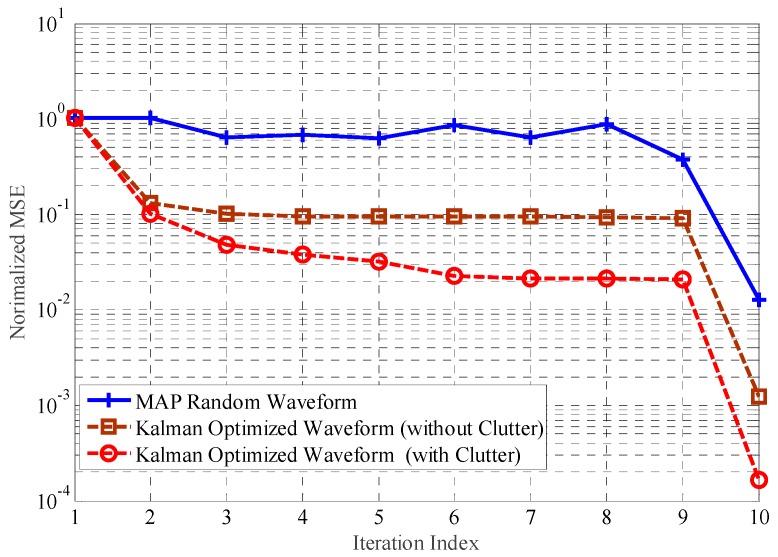
The estimation performance with the interference.

Secondly, we assume that the backscattering signals are disturbed by the clutter. We compare the performance gain of TIR estimation for optimization waveforms designed by the proposed scheme to the gain for random waveform, and compare this result with the optimized waveform designed by the proposed scheme, without considering the clutter. Figure 4 shows the normalized MSE with regard to TIR estimation.

Similarly, as shown in Figure 4, the performance of TIR estimation for optimization waveforms designed by the proposed scheme is improved, compared to the MAP criterion. Compared to the proposed scheme considering the clutter, the performance gain offered by the proposed scheme is reduced, without considering the clutter. This result demonstrates that performance enhancement can be obtained, while interference factor is disregarded during waveform design process.

**Figure 5:**
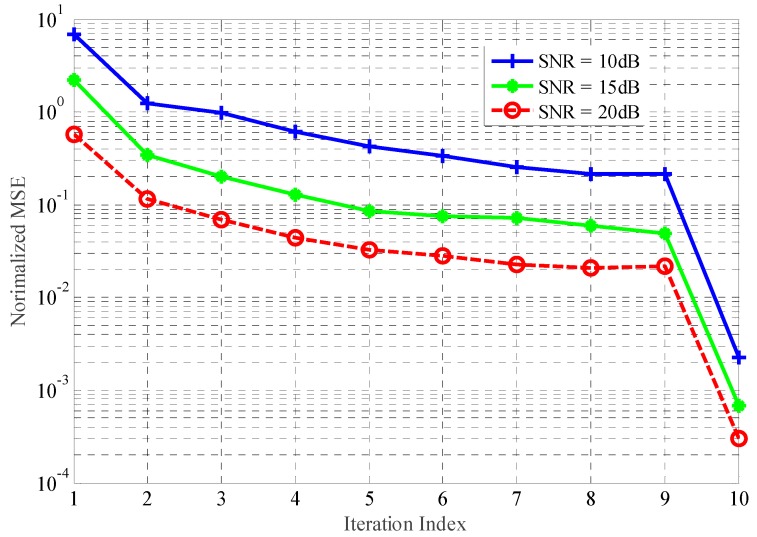
The estimation performance with different SNR.

**Figure 6:**
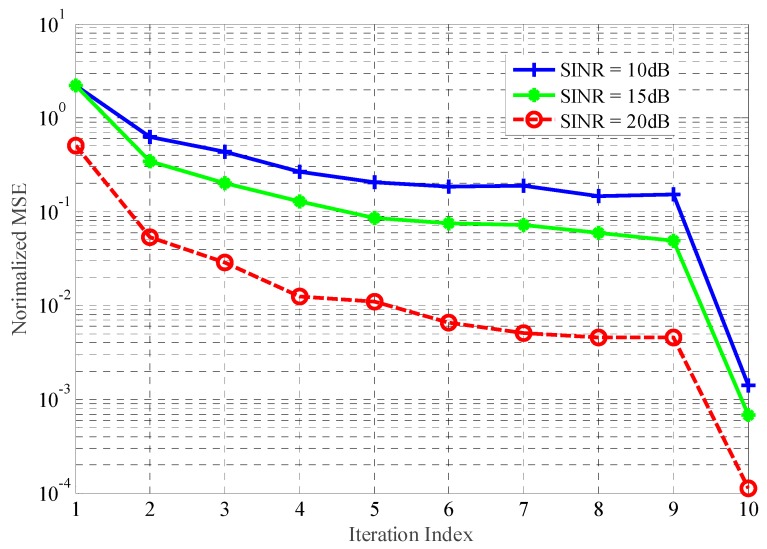
The estimation performance with different SINR.

Figure 5 and Figure 6 indicate the normalized MSE with regard to TIR estimation achieved by Kalman filtering approach under the transmitted power and detection probability constraints. Eight hundred simulations have been run for each, at a particular value of the received SNR and SINR, respectively. From the above figures, the performance gains in terms of TIR estimation achieved by the proposed method become larger with an increase in the SNR and SINR of received signals. In addition, the performance improvement in jammed environments is more efficient than that in the AWGN channel. The target feature information derived from successive received signals can be extracted, allowing the Kalman filtering approach to take full advantage of the temporal correlation of the TIR.

## 6. Conclusions

In this paper, we have developed a novel waveform design algorithm for adaptive multiple antennas radar system, which can improve the performance of TIR estimation. The receiver adopts the Kalman filtering approach by updating the target parameters. An adaptive feedback loop enables the delivery of the estimated value of TIR to the transmitter. The radar system updates the TIR estimation and utilizes this information to choose the optimal waveform for transmission. Finally, the simulation results show that the proposed scheme provides higher performance gains in terms of TIR estimation for multiple antenna scenarios under the transmitted power and detection probability constraints. As compared with the waveform design algorithm for TIR estimation based on MAP criterion, the proposed approach has relatively lower computational complexity. The next step will focus on the tradeoff between the complexity of computing and the performance improvement offered by the scheme.

## Figures and Tables

**Table 1:** Simulation Parameters.

Simulation Parameters		
$E_{f}$	Transmitted power	1
L	Length of transmitted signal	60
SNR	SNR	15 dB
λ	Temporal correlation	0.1 s
T	Pulse interval	1 ms
SINR	SINR	15 dB
$p_{fa}$	False alarm probability	0.01
$p_{d}$	Detection probability	0.95
N×Q	The number of the antennas	2×2
